# Correlates of Residual Thrombus Burden in Successfully Thrombolysed Patients of ST-Elevation Myocardial Infarction Receiving Dual Anti-Platelet Therapy

**DOI:** 10.7759/cureus.12017

**Published:** 2020-12-10

**Authors:** Parminder S Otaal, Abhinav Anand, Rajesh Vijayvergiya

**Affiliations:** 1 Cardiology, Post Graduate Institute of Medical Education and Research (PGIMER), Chandigarh, IND; 2 Internal Medicine, Post Graduate Institute of Medical Education and Research (PGIMER), Chandigarh, IND

**Keywords:** residual thrombus burden, stemi, thrombolysis, dual antiplatelet therapy

## Abstract

Background

Variable residual thrombus ranging from minimal to a large thrombus is seen in the culprit vessel after successful thrombolysis in ST-elevation myocardial infarction (STEMI). Factors associated with residual thrombus in thrombolysed patients are poorly understood. The objective of our study was to determine the correlates of residual thrombus burden in successfully thrombolysed STEMI patients receiving dual antiplatelet therapy.

Methods

In this prospective observational study of 60 successfully thrombolysed STEMI patients receiving dual antiplatelet therapy, various clinical and coronary angiographic features like residual thrombus burden, residual stenosis, and thrombolysis in myocardial infarction (TIMI) flow grade in the infarct-related artery were evaluated.

Results

Out of 60 patients, 49 and 11 patients, respectively, had low and high thrombus burden. Thirty-seven (75.5%) patients amongst low-grade thrombus had TIMI 3 flow, whereas seven (63.6%) amongst high thrombus burden had TIMI 2 flow indicating an association between residual thrombus burden and TIMI flow grade, which was statistically significant (p=0.009). Further, amongst the 39 patients who were 45 years old, a statistically significant association of age and residual stenosis (p = 0.039) was observed.

Conclusion

In successfully thrombolysed STEMI patients receiving dual antiplatelet therapy, there is an inverse correlation between residual thrombus burden and TIMI flow grade with high-grade residual thrombus associated with more frequent low TIMI flow. Further, significant residual stenosis is more common in patients older than 45 years of age, underscoring the necessity for invasive evaluation after successful thrombolysis.

## Introduction

Coronary artery disease (CAD) is the leading cause of mortality and morbidity all over the world. Over the past two decades, there has been an alarming increase in the prevalence of CAD and cardiovascular mortality in India and other South Asian countries [1]. According to a study on the effect of potentially modifiable risk factors associated with myocardial infarction in 52 countries (the INTERHEART study), the median age of the first heart attack at 53 years in Indians is a decade lower than in western Europe, Hong-Kong, and China with more men commonly affected than women. Half of the cardiovascular disease mortality in India occurs below the age of 50 years and one-fourth of acute myocardial infarction (MI) occurs below the age of 40 years [2]. Rupture of an atherosclerotic plaque and activation of the coagulation cascade followed by the formation of occlusive thrombus is the common pathogenesis of acute STEMI. Despite clinically successful thrombolysis, thrombus resolves usually partially or sometimes completely. ST-segment resolution on electrocardiogram (ECG) post thrombolysis is the simplest tool to assess coronary reperfusion in STEMI [3].

Even with the use of dual antiplatelet therapy in addition to thrombolysis, a residual thrombus is commonly seen even in patients achieving TIMI flow grade 3. Multivariate analysis has shown monocyte count to be an independent predictor of angiographic high-thrombus burden (p = 0.020) [4]. This residual thrombus may be a predictor of re-occlusion and major adverse coronary events, as well as stent thrombosis during and after percutaneous coronary intervention (PCI), thus contribute to morbidity and mortality. In addition, as older patients with STEMI are more likely to have underlying atherosclerotic stenosis in the coronary arteries, thrombus may have a greater relative contribution to coronary occlusion in younger counterparts and this may impact the residual thrombus and/or stenosis after successful thrombolysis. We also know that as the time to thrombolysis increases due to the gradual increase in the fibrin content with subsequent crosslinking and stabilization, the thrombus becomes more resistant to the action of thrombolytic agents. So thrombolytic therapy becomes less effective as time lapses after the onset of STEMI and this time delay may have an effect on residual thrombus burden. Further, the impact of the routine usage of dual antiplatelet therapy in patients undergoing successful thrombolysis on residual thrombus also remains largely unknown. Considering these variations, the objective of our study was to determine the factors that affect the residual thrombus burden in successfully thrombolysed patients in the current era of routine use of dual antiplatelet therapy.

## Materials and methods

This descriptive hospital-based prospective observational study was carried out at the Post Graduate Institute of Medical Education Research, Chandigarh, a tertiary care institute in Northern India. Patients of either sex, aged 18 years or older, with newly diagnosed STEMI who were thrombolysed with streptokinase and treated with dual antiplatelet therapy (both aspirin and clopidogrel) in addition to standard therapy were included in the study between January 2015 and May 2016. Patients with pre-existing noncardiac co-morbidities like previous MI, arrhythmia, congenital heart disease, or cardiomyopathy as well as patients in shock (systolic blood pressure <90 mmHg or mean arterial pressure <60 mmHg) were excluded from the study.

Patients were thrombolysed with streptokinase (dose 1.5 million units intravenous infusion) and received tablet aspirin 325 mg loading dose followed by 150 mg once a day orally and tablet clopidogrel 300 mg loading dose followed by 75 mg once a day orally. Patients received standard treatment for STEMI, including nitrate, atorvastatin, angiotensin-converting-enzyme (ACE) inhibitors, beta-blockers, unfractionated heparin (UFH)-low molecular weight heparin (LMWH), oxygen if saturation was less than 90%, all in standard doses unless contraindicated. ECG was accessed for the confirmation of successful thrombolysis. Coronary angiography was done to access TIMI flow grade (0,1,2,3) [5], residual thrombus burden, and residual stenosis in the infarct-related artery. Residual thrombus burden was assessed by using the TIMI thrombus grading system [6] with the reclassification of thrombus grade 0,1,2,3 as low-grade thrombus burden and 4,5 as high-grade thrombus burden [7]. The patients were managed according to standard protocols as per the American College of Cardiology Foundation/American Heart Association (ACCF/AHA) guidelines for the management of STEMI. However, a delay in time to angiography from the onset of pain could not be prevented due to late presentation. Since 45 years has been used in most of the studies to define young patients with CAD, the same was used as the cut-off between young and aged patients in this study as well.

The study was approved by our Institutional Ethical Convener, Thesis Committee (Approval Number: 9899/PG-2Trg/2014/5697-98) and conducted according to the ethical principles stated in the latest version of the Helsinki Declaration and the applicable guidelines for good clinical practice (GCP). Written informed consent was obtained from all the participants. The measurable data were checked for their normality using the Shapiro-Wilk test or the one-sample Kolmogorov-Smirnov test. Continuous variables, such as age and time, are presented as mean ± SD or median (inter-quartile range). Categorical variables, such as thrombus grade, TIMI flow, age (≤ 45 and > 45 years), and time to thrombolysis (≤ 3 and > 3 hours), are presented as percentages. The independent sample t-test was applied for between-group comparisons of normally distributed data, whereas non-normally distributed data were compared using the Mann-Whitney U test. The chi-square test or Fisher’s exact test, whichever was applicable, was applied for categorical variables. A probability value of ≤ 0.05 was accepted as statistically significant.

## Results

**Figure 1:**
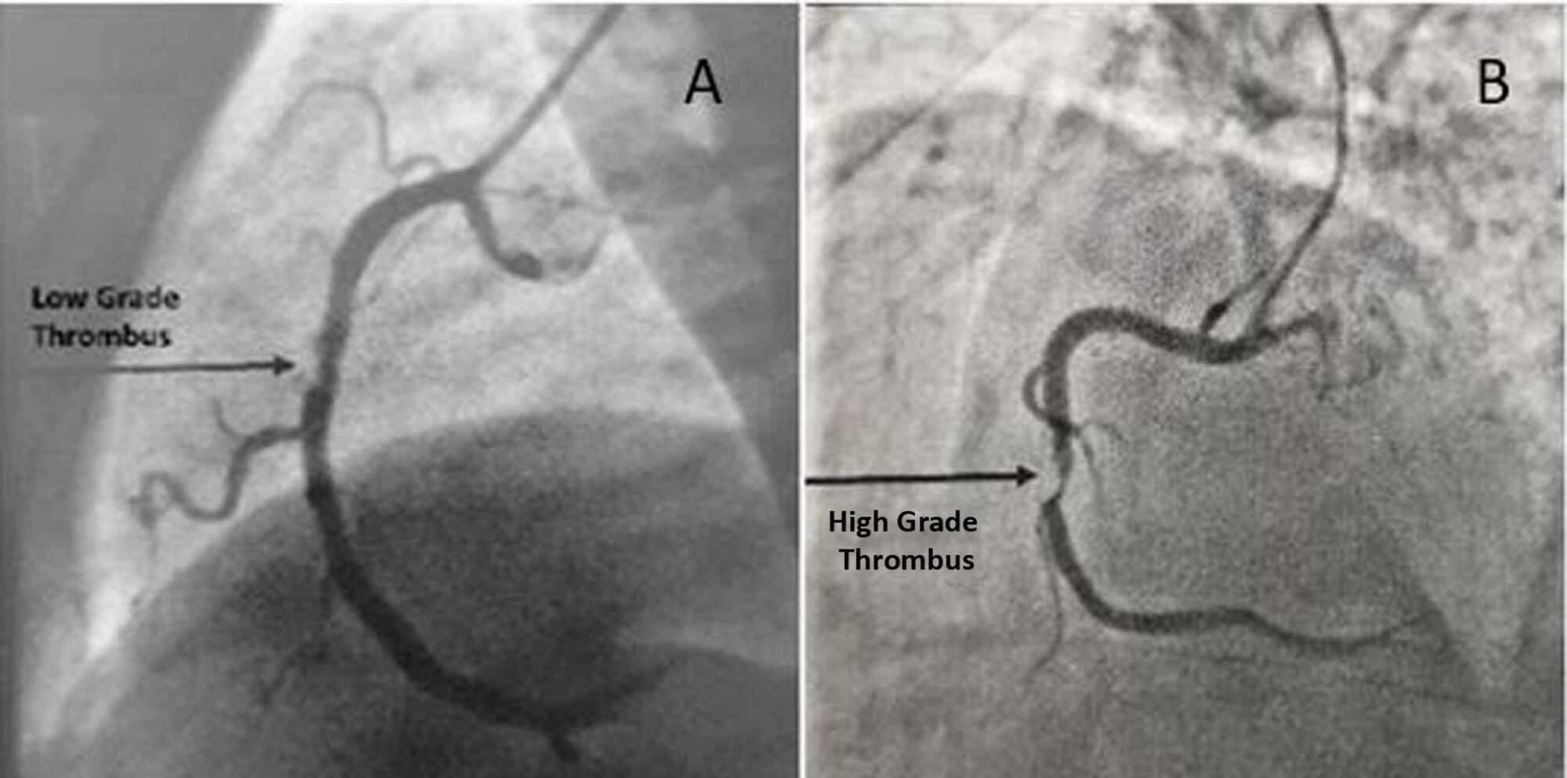
Coronary angiogram showing low-grade (A) and high-grade (B) thrombus

Sixty newly diagnosed (50 males and 10 females) successfully thrombolysed STEMI patients underwent coronary angiography to assess the infarct-related artery for residual thrombus burden, TIMI flow, and underlying stenosis. The study population was divided into a low thrombus burden group (n=49) and a high thrombus burden group (n=11) based on angiographic residual thrombus grade. Low-grade thrombus (Figure 1, panel 1A) included TIMI thrombus grades 0-3, whereas high-grade thrombus (Figure 1, panel 1B) included TIMI thrombus grades 4-5.

Baseline patient characteristics

Baseline characteristics were matched between the two groups. Although hypertension was more frequent in patients with high-grade thrombus, it was not a significant variable (Table 1).

**Table 1:** Baseline patient characteristics IQR: interquartile range; BMI: body mass index

S. No.	Parameter	Low grade thrombus (n=49)	High grade thrombus (n=11)	p value
1	Age, years (mean ± SD)	50.57 ± 12.44	55.64 ± 8.99	0.208
2	Time to thrombolysis (hours), median (IQR)	4 (3)	4.5 (4.5)	0.378
3	Diabetes, n (%)	13 (26.5%)	4 (36.4%)	0.712
4	Smoking, n (%)	24 (49.0%)	2 (18.2%)	0.093
5	Hypertension, n (%)	11 (22.4%)	6 (54.5%)	0.059
6	BMI ≥25 kg/m^2^, n (%)	18 (36.7%)	5 (45.5%)	0.734

Of the sixty patients, 21 (35%) were ≤ 45 years of age and 39 (65%) were > 45 years of age. Twenty-three (38.3%) patients were thrombolysed within three hours, whereas 37 (61.7%) patients were thrombolysed after three hours of the onset of chest pain.

Angiographic characteristics

The culprit artery for myocardial infarction was the left anterior descending (LAD) in 28 (46.7%) cases, the right coronary artery (RCA) in 27 (45%) cases, and the left circumflex (LCX) in five (8.3%) cases. Forty-nine (81.7%) patients had significant residual stenosis (stenosis ≥70%), whereas 11 (18.3%) patients had <70% stenosis post thrombolysis. All patients with a high-grade thrombus had significant stenosis as compared to three-fourth of patients with low-grade thrombus, although the difference was not significant. TIMI 3 flow was seen in 40 (66.7%) patients, whereas TIMI 2 and TIMI 1 flow was seen in 19 (31.7%) patients and one (1.7%) patient, respectively (Table 2).

**Table 2:** Angiographic characteristics ^#^Analysis did not include the LCX and OM arteries due to the small number of patients in the group ^##^Analysis did not include TIMI 1 due to the small number of patients in the group LAD: left anterior descending; RCA: right coronary artery; LCX: left circumflex; TIMI: thrombolysis in myocardial infarction

S. No.	Parameter	Low-grade thrombus (n=49)	High-grade thrombus (n=11)	P-value
1.	Infarct-related artery, n (%)			0.503^#^
	LAD	24 (49.0%)	4 (36.4%)	
	RCA	21 (42.9%)	6 (54.5%)	
	LCX	4 (8.2%)	1 (9.1%)	
2.	Residual stenosis	38 (77.6%)	11 (100%)	0.189
3.	TIMI flow, n (%)			0.009^##^
	1	-	1 (9.1 %)	
	2	12 (24.5 %)	7 (63.6 %)	
	3	37 (75.5 %)	3 (27.3 %)	

Association of residual thrombus burden

Thrombus Burden and TIMI Flow

Fischer’s exact test was performed to examine the relationship between residual thrombus burden and TIMI flow in the infarct-related artery. Out of 19 patients with TIMI grade 2 flow, seven had high-grade thrombus, whereas, amongst 40 patients with TIMI grade 3 flow, 37 had a low-grade thrombus burden on angiography. There was a statistically significant association between residual thrombus burden and TIMI flow grade (p=0.009). Most of the patients with low-grade thrombus had TIMI 3 flow and those with high-grade thrombus had TIMI 2 flow as shown in Table 3.

**Table 3:** Correlates of residual thrombus burden/residual stenosis * Significant value TIMI: thrombolysis in myocardial infarction

Residual Thrombus Burden				
	Low Grade	High Grade	Total	p-value
TIMI Flow				
TIMI flow grade 2	12 (20.3%)	7 (11.9%)	19 (32.2%)	0.009*
TIMI flow grade 3	37(62.7%)	3 (5.1%)	40 (67.8%)	
Total (n=59)	49 (83.1%)	10 (16.9%)	59 (100%)	
Time to Thrombolysis				
Duration ≤ 3 hours	21 (35%)	2 (3.3%)	23 (38.3%)	0.178
Duration > 3 hours	28 (46.7%)	9 (15%)	37 (61.7%)	
Total (n=60)	49 (81.7%)	11 (18.3%)	60 (100%)	
Residual Stenosis				
Age Groups	<70% stenosis	≥70% stenosis	Total	p-value
Age ≤45 years	7(11.7%)	14(23.3%)	21(35.0%)	0.039*
Age >45 years	4(6.7%)	35(58.3%)	39(65,0%)	
Total (n=60)	11(18.3%)	49(81.7%)	60(100%)	
Time to Thrombolysis				
Duration ≤ 3 hours	4 (6.7%)	19 (31.7%)	23 (38.3%)	1.0
Duration > 3 hours	7(11.7%)	30 (50.0%)	37 (61.7%)	
Total (n=60)	11(18.3%)	49 (81.7%)	60 (100%)	

Thrombus Burden and Time to Thrombolysis

**Figure 2:**
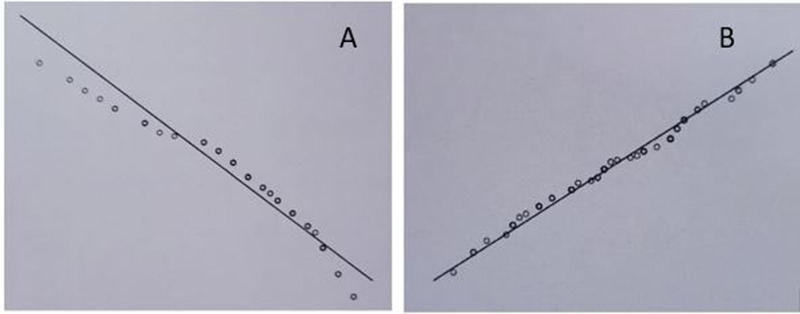
Q-Q plot for time to thrombolysis (A) and for age (B)

Fischer’s exact test was performed to examine the relationship between residual thrombus burden and time to thrombolysis. Out of 23 patients with a duration of chest pain prior to thrombolysis of ≤ 3 hours, 21 had a low thrombus burden. Amongst the remaining 37 patients who were thrombolysed after three hours of pain onset, 28 had a low thrombus burden on angiography. However, there was no statistically significant association between residual thrombus burden and time to thrombolysis (p=0.178) as shown in Table 3. As the data for the exact time duration of chest pain before thrombolysis was not normally distributed as assessed by the Shapiro-Wilk test (p=0.001) (Figure 2, panel 2A), the Mann- Whitney U test was used to determine the significant difference in the duration of chest pain in patients with a low- and high-grade thrombus burden. The result of the analysis indicated that there was no significant difference in the duration of chest pain between the low thrombus burden group (mean rank = 29.56) and the high thrombus burden group (mean rank = 34.68), (U = 315.5, z = 0.881, p=0.378).

Thrombus Burden and Time to Angiography

The median time to angiography from the onset of pain in patients with a low-grade and high-grade thrombus burden was 42 hours and 44 hours respectively, the delay being due to late presentation. Mann-Whitney U test was used to determine the significant difference in the time for angiography in patients with low- and high-grade thrombus burden. Results of the analysis indicate that there was no significant difference in time for angiography in the low thrombus burden group (mean rank =30.31) and the high thrombus burden group (mean rank =31.36), (U = 279, z = 0.182, p = 0.856).

Thrombus Burden and Age

Fischer’s exact test was performed to examine the relationship between the residual thrombus burden and the age of the patient. Out of 21 patients of age ≤ 45 years, 20 had a low-grade thrombus burden. Amongst the remaining 39 patients of age > 45 years, 29 had a low-grade thrombus burden on angiography. However, there was no statistically significant association between the residual thrombus burden and the age of the patient (p=0.078). Further, an independent sample t-test was done to determine any significant age difference in patients with a low and high thrombus burden. There was no outlier in the data as assessed by the inspection of the box-plot. The normality of the data was assessed with the Shapiro-Wilk test (p=0.356); inspection of Q-Q plots reveals that age was normally distributed (Figure 2, panel 2B).

There was homogeneity of variance as assessed by Levene’s test for equality of variance. The study found that there was no significant difference in the mean age of patients with high angiographic residual thrombus burden (55.64 ± 8.99 years) as compared to patients with a low thrombus burden (50.57 ± 12.44), t(58)= -1.274, p=0.208.

Associations of residual stenosis

Residual Stenosis and Age

Fischer’s exact time test was performed to evaluate the relationship between significant residual stenosis in the infarct-related artery and the age of the patients. Out of 21 patients of age ≤ 45 years, 14 had > 70% stenosis, whereas, amongst 39 patients who were > 45 years old, 35 had ≥ 70% stenosis on angiography. There was a statistically significant association between patients' age and residual stenosis (p = 0.039), with significant residual stenosis (≥ 70 %) being significantly more common in patients > 45 years of age as shown in Table 3.

Residual Stenosis and Time to Thrombolysis

Fischer’s exact test was performed to evaluate the relationship between time to thrombolysis and residual stenosis in the infarct-related artery. As shown in Table 3, out of 23 patients with time to thrombolysis ≤ 3 hours, four had < 70 % residual stenosis, whereas out of 37 patients thrombolysed > 3 hours after onset of pain, 30 had > 70% stenosis on angiography. However, there was no statistically significant association between time to thrombolysis and residual stenosis (p = 1.00).

## Discussion

Correlation of residual thrombus burden and residual stenosis with patient’s age

The clinical and angiographic data in a cohort of 60 patients of successfully thrombolysed STEMI in our study had shown no statistically significant association between the age of the patients and residual angiographic thrombus burden post thrombolysis (p=0.078). Tanboga et al. had also evaluated factors that determine the angiographic thrombus burden in patients of MI undergoing primary PCI and concluded that there was no significant association between the age of patients and thrombus burden (high and low thrombus burden groups) (p=0.888) [8]. A number of other studies have classified thrombus into high and low-grade thrombus burden in the infarct-related artery and tried to distinctly define the factors that influence patients undergoing PCI [7,9-11]. Similar to our findings, none of the studies has established a significant association between thrombus burden grade and age. This may indirectly suggest that as far as the resolution of thrombus is concerned, current therapy is age independent and is equally efficacious in younger and older patients with STEMI.

However, various studies on the correlation of patients' age to residual stenosis including the one by Schweiger et al., observed that patients with <60% residual stenosis in the culprit artery were younger than patients with ≥60% stenosis [12]. Llevadot et al. also observed that patients with <50% residual stenosis were younger as compared to patients with ≥ 50% stenosis (p=0.02) [13]. The results of the Coronary Artery Surgery Study Registry (CASS) trial also showed that younger patients have either angiographically normal arteries or have mild obstruction [14]. These results are similar to the result of our study, which has also shown a significant association between the age of the patient and significant residual stenosis (p=0.025). Residual stenosis (≥70%) was significantly more common in patients older than 45 years of age in comparison to younger patients reflecting underlying pre-existing atherosclerotic occlusion of coronary arteries. Thus, our study reveals that in successfully thrombolysed STEMI patients, the residual stenosis and not the residual thrombus have a significant association with the patient’s age. This underscores the necessity for an invasive angiographic assessment of such patients even after successful thrombolysis, as significant residual stenosis is an indication for revascularization.

Correlation of residual thrombus burden and residual stenosis to time to thrombolysis

The significance of early treatment on the success of thrombolysis and mortality has been well-established in many trials. Although there is some data to suggest that aspiration thrombectomy is associated with a possible small decrease in mortality and a small increase in stroke [15], the routine use of aspiration thrombectomy in STEMI is not recommended and its role as a bailout procedure in a select group of patients with large thrombus burden needs to be evaluated. In a subset analyses of a study on clopidogrel as adjunctive reperfusion therapy including thrombolysed patients in MI (CLARITY-TIMI 28 study) who received dual antiplatelet therapy, Kirtane et al., did not find a significant difference between the time to thrombolysis and the presence and absence of thrombus [16]. Similarly, Yip et al. observed that there was no statistically significant difference in the incidence of high-grade thrombus burden in patients undergoing primary PCI in <4 hours versus >4 hours of the onset of symptoms (p=0.972) [17]. In another study, Youssef et al. showed that the difference in reperfusion time between the low and high thrombus group was not statistically significant (p=0.627) [10]. Nicolli et al., in their study on the effect of aspirin therapy on thrombus burden, also did not find a significant relationship between time to PCI and thrombus burden (p=0.430) [7]. Gatto et al., had, however, shown the possible correlation between residual intra-stent thrombus and angiographic myocardial reperfusion [18]. Although one expects a large thrombus burden in patients thrombolysed late after STEMI, our study surprisingly finds no statically significant association between the time to thrombolysis and residual thrombus burden (p=0.178) or residual stenosis (p>1.0). This may be partially contributed by the possibility that the routine use of dual antiplatelet therapy may help reduce thrombus burden in successfully thrombolysed STEMI patients by preventing propagation of thrombus.

The association of residual thrombus burden with TIMI flow, residual stenosis, and conventional risk factors for coronary artery disease

In the present study, a high-grade thrombus burden was associated with low-grade TIMI flow on angiography and vice-versa (p=0.009). Toutouzas et al. found similar results in their study of 59 patients who received dual antiplatelets and were thrombolysed with tenecteplase [19]. The patients with high-grade thrombus burden had less frequent TIMI 3 flow (p=0.02). Similarly, the results of the CLARITY TIMI 28 sub-study had shown that there was less frequent TIMI 3 flow in the presence of angiographically evident thrombus (p<0.001) [16]. Tanbogaet et al. and Kurt et al. found similar results in patients undergoing primary PCI [8-9]. In another study, Ahn et al. had found that patients with a presenting residual thrombus or a TIMI flow grade of up to 2 had higher no-reflow incidence than those with no visible pre-stenting thrombus and a TIMI flow grade of 3 (74 versus 6.2%, p<0.001) [20].

Our study did not show a significant association of thrombus burden with residual stenosis (p=0.183) or culprit artery involved (p=0.503). The risk factors for CAD (diabetes, hypertension, smoking, and obesity) did not differ between patients with high- and low-grade residual thrombus burden. Our study is unique in including only successfully thrombolysed patients, although findings of other studies assessing factors affecting low- and high-grade thrombus burden in patients undergoing primary PCI [8-9,11,21] also apply to successfully thrombolysed patients receiving dual antiplatelet therapy.

## Conclusions

In successfully thrombolysed STEMI patients receiving dual antiplatelet therapy, there is an inverse correlation between residual thrombus burden and TIMI flow grade with high-grade residual thrombus associated with more frequent low TIMI flow. Further, significant residual stenosis is more common in patients older than 45 years of age, underscoring the need for routine angiography to detect and treat significant residual stenosis after STEMI, which otherwise is an ACC/AHA class IIb indication. Additionally, traditional risk factors do not bear a significant correlation with thrombus burden.

## References

[1] Krishnan MN (2012). Coronary heart disease and risk factors in India - on the brink of an epidemic?. Indian Heart J.

[2] Dalal J, Sahoo PK, Singh RK (2013). Role of thrombolysis in reperfusion therapy for management of AMI: Indian scenario. Indian Heart J.

[3] van 't Hof AW, Liem A, de Boer MJ, Zijlstra F, Zwolle Myocardial infarction Study Group (1997). Clinical value of 12-lead electrocardiogram after successful reperfusion therapy for acute myocardial infarction. Lancet.

[4] Wang Z, Liu N, Ren L, Lei L, Ye H, Peng J (2018). Association of monocyte count on admission with the angiographic thrombus burden in patients with st-segment elevation myocardial infarction undergoing primary percutaneous coronary intervention. Arq Bras Cardiol.

[5] Gibson CM, Schömig A (2004). Coronary and myocardial angiography: angiographic assessment of both epicardial and myocardial perfusion. Circulation.

[6] Gibson CM, de Lemos JA, Murphy SA (2001). Combination therapy with abciximab reduces angiographically evident thrombus in acute myocardial infarction. A TIMI 14 substudy. Circulation.

[7] Niccoli G, Spaziani C, Marino M (2010). Effect of chronic aspirin therapy on angiographic thrombotic burden in patients admitted for a first ST-elevation myocardial infarction. Am J Cardiol.

[8] Tanboga IH, Topcu S, Aksakal E, Kalkan K, Sevimli S, Acikel M (2014). Determinants of angiographic thrombus burden in patients with ST-segment elevation myocardial infarction. Clin Appl Thromb Hemost.

[9] Kurt M, Karakas MF, Buyukkaya E, Akçay AB, Sen N (2014). Relation of angiographic thrombus burden with electrocardiographic grade III ischemia in patients with ST-segment elevation myocardial infarction. Clin Appl Thromb Hemost.

[10] Youssef AA, Chang LT, Sheu JJ (2007). Association between circulating level of CD40 ligand and angiographic morphologic features indicating high-burden thrombus formation in patients with acute myocardial infarction undergoing primary coronary intervention. Circ J.

[11] Hamur H, Duman H, Bakirci EM, Kucuksu Z, Demirelli S, Kalkan K, Degirmenci H (2016). Bilirubin levels and thrombus burden in patients with st-segment elevation myocardial infarction. Angiology.

[12] Schweiger MJ, McMahon RP, Terrin ML (1994). Comparison of patients with < 60% to > or = 60% diameter narrowing of the myocardial infarct-related artery after thrombolysis. The TIMI Investigators. Am J Cardiol.

[13] Llevadot J, Giugliano RP, McCabe CH (2000). Degree of residual stenosis in the culprit coronary artery after thrombolytic administration (thrombolysis in myocardial infarction [TIMI] trials). Am J Cardiol.

[14] Zimmerman FH, Cameron A, Fisher LD, Ng G (1995). Myocardial infarction in young adults: angiographic characterization, risk factors and prognosis (coronary artery surgery study registry). J Am Coll Cardiol.

[15] El Dib R, Spencer FA, Suzumura EA, Gomaa H, Kwong J, Guyatt GH, Vandvik PO (2016). Aspiration thrombectomy prior to percutaneous coronary intervention in ST-elevation myocardial infarction: a systematic review and meta-analysis. BMC Cardiovasc Disord.

[16] Kirtane AJ, Vafai JJ, Murphy SA, Aroesty JM, Sabatine MS, Cannon CP (2006). Angiographically evident thrombus following fibrinolytic therapy is associated with impaired myocardial perfusion in STEMI: a CLARITY-TIMI 28 substudy. Eur Heart J.

[17] Yip HK, Chen MC, Chang HW, Hang C-L, Hsieh Y-K, Fang C-Y, Wu C-J (2002). Angiographic morphologic features of infarct-related arteries and timely reperfusion in acute myocardial infarction. Predictors of slow-flow and no-reflow phenomenon. Chest.

[18] Gatto L, Romagnoli E, Versaci F (2017). The role of residual intrastent thrombus during primary angioplasty: insights from the COCTAIL II study. J Cardiovasc Med.

[19] Toutouzas K, Tsiamis E, Karanasos A (2010). Morphological characteristics of culprit atheromatic plaque are associated with coronary flow after thrombolytic therapy: new implications of optical coherence tomography from a multicenter study. JACC Cardiovasc Interv.

[20] Ahn SG, Choi HH, Lee JH (2015). The impact of initial and residual thrombus burden on the no-reflow phenomenon in patients with ST-segment elevation myocardial infarction. Coron Artery Dis.

[21] Duman H, Çetin M, Durakoğlugil ME (2015). Relation of angiographic thrombus burden with severity of coronary artery disease in patients with ST segment elevation myocardial infarction. Med Sci Monit.

